# Patient Perspectives on Improving Patient-Provider Relationships and Provider Communication During Opioid Tapering

**DOI:** 10.1007/s11606-021-07210-9

**Published:** 2022-01-06

**Authors:** Sarah Kosakowski, Allyn Benintendi, Pooja Lagisetty, Marc R. Larochelle, Amy S. B. Bohnert, Angela R. Bazzi

**Affiliations:** 1 Clinical Addiction Research and Evaluation Unit, Section of General Internal Medicine, Boston Medical Center, Boston, MA USA; 2 Michigan Medicine, Department of Internal Medicine, Ann Arbor, MI USA; 3 VA Center for Clinical Management Research, Ann Arbor, MI USA; 4 Michigan Medicine, Department of Anesthesiology, Ann Arbor, MI USA; 5 Herbert Wertheim School of Public Health and Human Longevity Science, University of California, La Jolla, CA USA; 6 Department of Community Health Sciences, Boston University School of Public Health, 801 Massachusetts Avenue, Boston, MA 02118 USA

**Keywords:** Analgesics, opioid, Chronic pain, Taper, Communication, Professional-patient relations

## Abstract

**Background:**

Efforts to reduce opioid overdose fatalities have resulted in tapering (i.e., reducing or discontinuing) opioid prescriptions despite a limited understanding of patients’ experiences.

**Objective:**

To explore patients’ perspectives on opioid taper experiences to ultimately improve taper processes and outcomes.

**Design:**

Qualitative study.

**Participants:**

Patients on long-term opioid therapy for chronic pain who had undergone a reduction of opioid daily prescribed dosage of ≥50% in the past 2 years in two distinct medical systems and regions.

**Approach:**

From 2019 to 2020, we conducted semi-structured interviews that were audio-recorded, transcribed, systematically coded, and analyzed to summarize the content and identify key themes regarding taper experiences overall and with particular attention to patient-provider relationships and provider communication during tapers.

**Key Results:**

Participants (*n*=41) had lived with chronic pain for an average of 17.4 years (range, 3–36 years) and described generally adverse experiences with opioid tapers, the initiation of which was not always adequately justified or explained to them. Consequences of tapers ranged from minor to substantial and included withdrawal, mobility issues, emotional distress, exacerbated mental health symptoms, and feelings of social stigmatization for which adequate supports were typically unavailable. Narratives highlighted the consequential role of patient-provider relationships throughout taper experiences, with most participants describing significant interpersonal challenges including poor provider communication and limited patient engagement in decision making. A few participants identified qualities of providers, relationships, and communication that fostered more positive taper experiences and outcomes.

**Conclusions:**

From patients’ perspectives, opioid tapers can produce significant physical, emotional, and social consequences, sometimes reducing trust and engagement in healthcare. Patient-provider relationships and communication influence patients’ perceptions of the quality and outcomes of opioid tapers. To improve patients’ experiences of opioid tapers, tapering plans should be based on individualized risk-benefit assessments and involve patient-centered approaches and improved provider communication.

**Supplementary Information:**

The online version contains supplementary material available at 10.1007/s11606-021-07210-9.

## INTRODUCTION

Higher opioid analgesic dosages are associated with increased rates of drug-related overdose morbidity and mortality.^1–3^ Furthermore, recent clinical trials comparing opioids to alternatives have raised questions about the effectiveness of opioids for chronic pain.^4,5^ In recent years, several federal agencies have published safe opioid-prescribing guidelines recommending tapering to reduce dosages or discontinue in some situations.^6–9^ The Centers for Disease Control and Prevention (CDC) released the Guideline for Prescribing Opioids for Chronic Pain in March 2016, which was associated with a reduced prevalence of higher opioid analgesic dosages.^1^

There is limited guidance about best practices to taper opioid analgesics. Reports of patient harms from abrupt opioid discontinuation prompted the CDC Guideline authors to clarify that evidence did not support abrupt opioid discontinuation and caution against this practice.^10^ State-level programs and initiatives to taper opioid dosages or set dosage limits have become more common; however, early evidence suggests only modest reductions in opioid prescribing, while broader impacts on opioid-related mortality, morbidity, and pain control remain unknown.^11–13^ Furthermore, a systematic review found that evidence on patients’ toleration of opioid tapering was of low quality and limited to trials with participants who voluntarily tapered,^14^ limiting the generalizability of findings.

Relative to the widespread implementation of opioid taper initiatives, patients’ experiences in this realm remain poorly understood. One 2016 study described the perceived risks and benefits of opioid tapering among individuals who were currently or previously taking prescribed opioids.^15^ Despite the small number of participants in this sample who had actually undergone opioid tapers (*n*=6), their perspectives hinted at a worrisome overall experience that has been highlighted in recent quantitative research linking opioid tapers to patients’ subsequent termination of care.^16^ There is a particular lack of information regarding patient perspectives on what could improve taper experiences and outcomes.

While published guidelines mention shared decision-making and centering patient goals within tapering plans, little specific direction is available in terms of how to implement these general recommendations despite emerging research suggesting a need for improved communication (e.g., by tailoring tapering messaging to patients based on individual circumstances and encouraging patient input during taper processes).^17^ Consequentially, providers have described challenges implementing tapers including poor perceptions of communication, emotional burden, lack of relational trust, and inadequate training and guidance.^18^

The challenges and potential unintended consequences of efforts to reduce opioid prescribing call for immediate attention to the experiences and needs of patients for whom opioid tapering interventions are intended to serve. We thus conducted an in-depth qualitative study to explore patients’ perspectives on their overall opioid taper experiences, including patient-provider relationships and provider communication during tapers, with the ultimate goal of improving the quality of care.

## METHODS

### Study Design and Sample

We recruited patients on long-term opioid therapy for chronic pain (hereafter “participants”) from primary care clinics at Boston Medical Center (BMC) in Boston, MA, and addiction treatment services, pain management, and primary care clinics, and the High Dose Opioid Tapering Initiative clinic at University of Michigan Health System (UMHS; https://anes-conf.med.umich.edu/opioidtaper/) in Ann Arbor, MI. We reviewed medical charts to assess eligibility, which included being an adult (≥18 years old) BMC/UMHS patient with a peak opioid daily prescribed dosage >50 morphine milligram equivalents [MME] between 1/1/2017 and 2/1/2020 and with current (as of the review date) opioid dosages ≥50% lower than peak dosages identified in charts, also as of the review date). We also received referrals from primary care physicians who supervised opioid tapers and reviewed referred individuals’ medical charts to confirm eligibility. We screened and obtained informed consent from all participants by phone prior to enrollment. Participants received $50 gift cards for participating. The BMC and UM Institutional Review Boards approved all study protocols.

### Data Collection

We collected data in person or by phone from August 2019 to February 2020. A lead, PhD-level qualitative investigator trained four master’s-level interviewers via an interactive workshop and provided ongoing supervision and feedback throughout data collection. Interviewers first administered brief quantitative assessments of socio-demographics (e.g., age, race/ethnicity, gender), time living with chronic pain, and opioid medication-related behaviors. Interviewers then used a semi-structured interview guide based on literature and our interdisciplinary team’s experience (including clinicians, researchers, and addiction medicine and chronic pain specialists) to explore participants’ histories of chronic pain and long-term opioid therapy, experiences of opioid tapers, and suggestions for how to improve taper implementation (see Appendix). Interviews were audio-recorded and lasted ~45 min. Interviewers were trained to administer the Columbia-Suicide Severity Rating Scale (C-SSRS)^19^ if suicidal ideations arose during interviews. Interviewers met weekly to discuss data collection progress.

### Data Analysis

Interview recordings were professionally transcribed and reviewed in weekly meetings to inform codebook development and analyses. About interviewing half of the target sample size, we initiated an iterative, collaborative codebook development process incorporating feedback from the entire team.^20,21^ We developed deductive codes based on key topics of interest from the interview guide (e.g., “reason for taper,” “taper process,” “patient-provider relationship”) and inductive codes (e.g., “emotional stress,” “mistrust in providers/system”) for emergent topics. Using selected transcript excerpts, we iteratively tested and refined codes to establish interpretive consensus and agree as a team that codes were adequately structured and defined. We then double-coded an initial set of full transcripts in NVivo to further evaluate codebook completeness and consistency of coding across analysts. A single analyst then coded the remaining transcripts in NVivo. From discussing key topics of interest that had been coded, and not observing new topics emerging, we determined as a team when we had attained thematic saturation and could cease recruiting new participants.^22^ Coded data were then analyzed alongside a review of the relevant medical and health sciences literature, with particular attention to participants’ opioid taper experiences. Key findings are illustrated using representative, anonymized quotes.

## RESULTS

Among 41 participants, age ranged from 28 to 76 years; over half identified as female (56%) and White (66%); nine (22%) identified as Black/African American and five as other or multiple races (12%; Table 1). Participants had lived with chronic pain for an average of 17.4 years (range, 3–36 years), which they related to various injuries and conditions including arthritis, Behcet’s disease, Crest syndrome, Crohn’s disease, diabetes, fibromyalgia, Grave’s disease, injuries, and sickle cell anemia. In brief quantitative assessments, over half (53%) of participants reported taking more of their opioid medications than prescribed and a quarter (26%) reported borrowing opioids from others.

**Table 1:** Characteristics of Patients with Chronic, Non-cancer Pain (*n*=41)

	**Boston Medical Center**	**Michigan Medicine**	**TOTAL**
***n* (%)**	9 (21.95)	32 (78.05)	41 (100)
**Age**			
25–44	2 (22.22)	11 (34.38)	13 (31.71)
45–54	1 (11.11)	8 (25.00)	9 (21.95)
55–64	3 (33.33)	8 (25.00)	11 (26.83)
65+	3 (33.33)	5 (15.62)	8 (19.51)
Race*			
American Indian/Alaska Native	0 (0)	1 (3.12)	1 (2.44)
Black/African American	4 (44.44)	5 (15.62)	9 (21.95)
White	3 (33.33)	24 (75.00)	27 (65.85)
Other	1 (11.11)	2 (6.25)	3 (7.32)
Mixed (>1 race)	1 (11.11)	0 (0)	1 (2.44)
**Gender**			
Male	4 (44.44)	14 (43.75)	18 (43.90)
Female	5 (55.56)	18 (56.25)	23 (56.10)
**Duration living with chronic condition**			
Years (mean ± sd)	17.7 (10.7)	17.3 (11.0)	17.4 (10.9)
**Took more opioid medication than prescribed**			
	5 (55.56)	17 (53.12)	22 (53.66)
**Borrowed opioid medication from someone else**			
	2 (22.22)	9 (28.12)	11 (26.83)

^*^Note: Asian and Pacific Islander and Ethnicity (Hispanic/Latino) had zero counts

In qualitative interviews, participants described their taper initiation circumstances and a range of physical, emotional, and social consequences. Most participants detailed significant interpersonal and communication challenges with providers; some provided suggestions for improved taper-related communication and more individualized planning. These findings are detailed in the sections below.

### Varying Taper Initiation Circumstances

Participants described their opioid tapers as being initiated by themselves or their providers. Participants who framed their tapers as self-initiated described their prior concerns about increasing physiologic dependence on opioids or possibly having a substance use disorder, decreased quality of life, and wanting to “reclaim control” over their lives and worry less about opioid access. Some of these participants did not want to “deal with” or “feel beholden to” opioid prescribers.

Conversely, participants viewed provider-initiated tapers as part of providers’ risk mitigation strategies, heightened institutional pressure to reduce opioid prescribing, and the “government cracking down.” Some of these participants felt that their providers were overly concerned about protecting their medical licenses or that their tapers had been policy-driven rather than based on their individual needs, as one participant reflected, “The Hippocratic Oath that’s supposed to protect the patient—‘Do no harm,’—that’s become, ‘Do no harm to the doctor’s license.’” Some participants explained that providers’ rationales were impersonal, insufficient, and even inaccurate, leading them to feel mislabeled as “[opioid] dependent” or having a substance use disorder. These participants reported being told briefly and generally that their dosages were “too high,” they were “too tolerant,” or that they had been prescribed opioids for “too long.”

When providers’ rationales for tapering were vague or absent, participants speculated about providers’ motivations. For some, this included broad opining about chronic pain treatment in the US “opioid epidemic” context: “They figure that if you’re on [opioids] past maybe a year or something, you’re pretty much addicted.” For others, unclear rationales resulted in more conspiratorial concerns that providers feared punishment or were even rewarded by external actors for tapering patients off of opioids. As one participant explained, his provider “wanted to get out [of opioid prescribing] because of the government.”

### Physical, Emotional, and Social Consequences

Participants experienced a range of immediate physical and emotional reactions to the initial phases of their opioid tapers. Physical reactions involved increased pain and symptoms of opioid withdrawal that were “miserable” and “excruciating,” involving nausea, vomiting, diarrhea, sweating, decreased hunger, sleeplessness, anxiety, and weight gain. Many also described heightened immobility during tapers, with one participant sharing, “I was in so much pain [that] I couldn’t do anything. I could not function. I could not go anywhere. I could not do anything but lie on a heating pad and cry and scream from the pain.” Following these early taper experiences, participants described lasting pain and inadequate pain relief that often went unaddressed by clinicians. When asked how his provider responded to his complaints of increased pain, one participant stated simply, “He didn’t care.”

Opioid tapers exacted significant mental health and emotional toll on some participants who described feeling “upset,” “worried,” “nervous,” “stressed,” “exasperated,” “angry,” “agitated,” and “worthless.” Other impacts to mental health included episodes of crying and panic attacks, as well as overall feelings of anxiety and sadness. Some described using alcohol to cope with the physical and emotional discomfort of the taper process, with one participant stating that during the taper, “I was trying to drink myself to death.” One participant described buying OxyContin “on the streets” when he could afford it. In some cases, self-harm and suicidal ideations were described as occurring during previous opioid tapers (however, these experiences were not related to recent or continuing tapers, and the C-SSRS protocol was only initiated once, resulting in an assessment of “low risk”). Nevertheless, some participants used language related to death and dying to express the severity of their taper experiences.

Compounding the physical and emotional reactions to tapers, many participants also felt socially stigmatized during the taper process, believing that their providers viewed them as “drug-seeking” or being a “junkie” or “addict,” leading to feelings of “shame,” “guilt,” and “humiliation.” Furthermore, participants reported awareness of and mixed attitudes towards providers’ efforts to monitor their opioid use surrounding their tapers (e.g., through pill counts), causing them to feel highly scrutinized, judged, and mistrusted. These physical, emotional, and social tolls of opioid tapers were described as being interconnected, with one participant linking their emotional distress with increased pain: “When I get very anxious, when I get very upset, my pain does get worse; it’s directly related.” Others explained how their physical health challenges directly exacerbated their mental health conditions, and vice versa. Thus, opioid tapers represented periods of acute physical, emotional, and social challenges for most participants.

### Significant Interpersonal and Communication Challenges with Providers

Related to the challenges described above, many participants expressed confusion and frustration with respect to their providers’ interactions and communications with them, which were often sparing or unclear. This included initial conversations about tapers (i.e., prior to taper initiation), which were especially fraught in some cases. When asked about the reason for her taper, one participant shared, “[My provider] wouldn’t say anything. I was just greeted with silence…the attitude was, ‘Well, tough, this is all I can give you now.’” Others were frustrated to learn about opioid tapers through impersonal voicemails or letters, without any opportunity to discuss taper processes with providers, ask questions, or express concerns. One participant only learned about his opioid taper during a routine pharmacy visit when his opioid prescription was no longer available.

Following taper initiation, communication challenges often persisted, with participants desiring greater discussion with providers and often feeling confused about key elements of the process. When attempting to notify providers of their increased pain and other symptoms, several participants described conversations that made them feel “misunderstood,” “dismissed,” “unheard,” or “ghosted.” Some described tailoring their own communication style in an attempt to be understood or viewed more positively by providers, as one participant explained, “I’ve always kept it casual because you’re afraid of their judgments…You always have that fear of, are they going to believe you, or are they going to think that you just want to stay at these higher doses...so I keep it low-key.” As described above, of the conversations that did occur with providers, many were described as “abrupt” and even stigmatizing.

After their tapers, some participants decided to not return to the providers who tapered their opioids and instead sought care elsewhere. They reported compromised trust due to poor communication, feeling unheard or disbelieved, perceiving their taper to be a non-consensual process, or viewing it as yet another example of their struggles to maintain stable healthcare. As one participant explained, “I don’t see that doctor anymore because, like I said, I don’t trust him. I’m not even pleased with the doctor I see currently; I don’t trust her either.” Following tapers, some participants described long periods elapsing since seeking any healthcare (e.g., “over a year”) while others had not returned to the healthcare system at all (e.g., “I’ve never been back”). One participant reported that the poor communication during his taper experience had caused him to lose trust in the healthcare system and not return despite serious ongoing health challenges.

### Patient Preferences for Improved Taper-Related Communication and Planning

When asked how taper processes could be improved, many participants discussed the importance of providers engaging in clear, specific, non-stigmatizing, and “open communication” with patients. Specific recommendations included clearly explaining the entire tapering process prior to taper initiation and then being “patient,” “understanding,” “honest,” and available throughout the entire process. Some recommended that providers avoid confusing patients with chronic pain (i.e., who benefit from opioids) as “victims” of the “opioid crisis,” as one explained: “This whole opioid crisis stuff is really killing people that actually need pain management for legitimate reasons, and I think a lot of doctors are looking at that kind of stuff more than they are looking at how much pain somebody is in.” Relatedly, several participants recommended that providers discuss pain management “alternatives” to opioids that could be helpful during tapers.

Although many participants called for more in-depth conversations with providers before and during tapers, some acknowledged that affording time for this additional, improved communication could be difficult. Nevertheless, participants articulated how the plans for their tapers had been described only briefly and in ways that felt overly generic. To this point, one participant said, “They had a very set algorithm of the way they did things,” and called for more careful tailoring of taper plans to individuals’ unique circumstances and needs. One participant who recommended using written taper plans containing individualized goals and expectations echoed this sentiment by stating that, “nobody’s pain is the same.” Another participant elaborated, “[Patients] might have some common traits, but everybody’s different. What might work for one person may not work for [another].” As a final recommendation specific to communication, participants attempting to coordinate their care across multiple providers called for improved communication across clinical care teams to get providers “all on the same page.” As one participant explained, “There really wasn’t a lot of communication between the two [providers]. It was mostly me doing the communication between them…Everybody says they’re all working as a team, but sometimes they’re not.”

A minority of participants in our sample had positive taper experiences that highlight additional lessons for improving patients’ experiences. In addition to open communication, positive taper experiences were facilitated by flexibility regarding the pace and timeline of the taper, clear presentation of alternative pain management options, and specific provider qualities. Participants felt especially supported when their providers were “accessible” and responsive to multiple avenues of communication including patient portal messaging systems and phone calls. As one participant stated, “They’re very upfront with me about everything…about the tapering, and [with regard to the process] they explain ‘why.’” Additional provider qualities that facilitated positive taper experiences included “patience,” “gentleness,” “persistence,” and “empathy,” with two participants commenting on how their providers were “supportive” and provided “validation” of their pain and other concerns throughout taper processes. One participant with a positive experience shared that, “Having doctors who are there to champion you makes all the difference in the world.” Nevertheless, the narrative of another participant with a positive experience and highly supportive provider illuminated a gap in taper-related care regarding emotional wellbeing for which none of our participants had specific recommendations:

I would not be here if it wasn't for him, for more than one reason. His constant support through all of this is one of those [reasons], too. We need more doctors like that who actually deal with pain management, because that's the kind of support most doctors don't have to offer. But yet, the emotional support that comes into pain management, I think there's still a big, big empty space where there should be more of that.

## DISCUSSION

Despite increased efforts surrounding opioid tapering in clinical settings, emerging research suggests that opioid tapers may result in unintended consequences and negative downstream effects for patients.^16,23^ As patients’ perspectives on specific aspects of their opioid taper experiences had not been thoroughly described in existing literature, we conducted qualitative interviews with patients living with chronic pain in two distinct medical systems and regions who had undergone opioid tapers. Consistent with literature describing patient experiences with opioid prescribing and pain care more generally, our participants described experiencing significant physical, emotional, and social consequences of their opioid tapers that included withdrawal, mobility issues, emotional distress and mental health symptoms, and feelings of social stigmatization. Participants reported significant challenges with clinical providers, citing inadequate communication and generic or unclear tapering justifications and plans that impacted their therapeutic relationships with providers and resulted in feelings of distrust, betrayal, and abandonment. Given the potential harms of fractured patient-provider relationships prompted by tapering, our findings demonstrate the need for flexible, patient-centered opioid tapering processes with particular attention toward improved communication.

Many of our participants described a lack of clarity surrounding the reasons or justifications for their opioid tapers, as well as stigmatizing attitudes and actions throughout taper processes,^24^ and disagreements with providers in some cases. Based on our findings and published guidelines from federal agencies, tapering plans should be based on careful risk-benefit assessments with clear communication to patients prior to taper initiation.^6–9^ However, participants in our study expressed a desire for improved communication and engagement in taper planning and implementation, suggesting that risk-benefit assessments may not be occurring, or could be better leveraged as a tool to explain taper rationales (including objective reasoning) and engage patients in detailed discussions around tapering plans. Research could explore the extent to which this promotes patient buy-in by allowing patients to share their perceived risks and benefits of opioid use and concerns related to tapering prior to and during taper processes.^9^

Our study also showed that some participants felt that their tapers were policy-driven and wanted providers to offer more individualized planning involving more in-depth conversations around tapering motivations, plans, and pain management alternatives that were unique to individuals’ circumstances (i.e., rather than more generic rationales regarding institutional or government prescribing policies). A systematic review found that when providers facilitated shared-decision making processes with patients, their satisfaction, treatment adherence, and knowledge regarding treatment options increased.^25^ Another systematic review found that when there was buy-in from patients who agreed to opioid tapers, health outcomes such as function, pain, and quality of life improved, underscoring the need for patient input and bidirectional communication on individual circumstances throughout taper processes.^14^ Developing individualized plans could help patients understand the reasons for tapers while addressing perceived stigma and mental health concerns. Participants in our study noted a wide range of physical, mental, and behavioral health symptoms during tapers, including opioid withdrawal and negative changes in mood. It is critical for patients to feel that their providers are supportive and accessible throughout tapers, specifically when patients are experiencing difficult physical or mental health symptoms.^15,17,26^ Research could evaluate the impact of regular check-ins with patients as they are undergoing tapers to ensure that the tapering rate and pace are appropriate and any symptoms to the taper are being discussed and addressed.

Our participants and those in another study emphasized the importance of provider empathy and validation of patients’ concerns for building rapport and maintaining positive patient-provider relationships.^27^ Using patient perspectives described here, intervention research could develop and evaluate provider training initiatives focused on cultivating practical skills for engaging in patient-centered tapering conversations. Provider training materials could include sample scripts and interactive role-plays with feedback and training on effective communication strategies such as expressing understanding and compassion, validating patients’ experiences, and addressing patients’ concerns and questions. Motivational interviewing and consensus-building are potential techniques that could help providers and patients collaboratively set goals around opioid tapers.^28^ Some participants also reported that they felt dismissed by providers during their tapers, reducing their trust in providers and medical systems, and suggesting a need for taper communication to include assurances to patients that they will not be abandoned and will not experience tapers alone.^17^ As some of our participants described the psychological impacts of tapers, and literature suggests depressive symptoms are associated with increased risk of drop-out and relapse,^31,32^ collaborative care services (e.g., including mental health services primary care teams) could be helpful in retaining opioid taper patients in healthcare.

Our study has several limitations. Firstly, it was conducted at two academic medical centers with expertise in treating substance use disorders and/or chronic pain, limiting generalizability. However, the experiences patients shared were diverse and were not limited to our research sites. Secondly, due to literature gaps, we focused on patient experiences rather than provider perspectives, which should be elicited in subsequent research (particularly studies developing provider training interventions). Thirdly, recalling details of past tapers may have been challenging for some participants, although interviewers had lists of probes to help elicit details when necessary (see Appendix). Fourthly, patients often situated their taper experiences in wider contexts and shared general attitudes and perspectives pertaining to their chronic pain care, which may have confounded our results on specific taper experiences. Finally, our study was exploratory and was not designed to assess differences in experiences and outcomes between patients with self- vs. provider-initiated tapers or those receiving best-practice care vs. other taper approaches. Future qualitative, quantitative, and mixed-methods studies could engage larger, more diverse samples of patients and providers from different (including rural) regions and institutions to explore the impact of taper initiation circumstances on experiences and outcomes and develop and evaluate the effectiveness of specific intervention strategies to promote more best-practice care and patient-centered approaches to opioid tapering.

## CONCLUSION

Patients undergoing opioid tapers may experience a range of physical, social, and emotional challenges. Our study identified patient-provider relationships and communication as critical components of individuals’ opioid taper experiences. Improved communication and engagement of patients in their own taper planning, execution, and follow-up may improve patient experiences and overall wellbeing during and after opioid tapering.

## Supplementary Information

ESM 1 (DOCX 25.5 kb)

## References

[1] Bohnert ASB (2011). Association Between Opioid Prescribing Patterns and Opioid Overdose-Related Deaths. JAMA..

[2] Dunn KM (2010). Opioid Prescriptions for Chronic Pain and Overdose: A Cohort Study. Ann Intern Med..

[3] Gomes T, Mamdani MM, Dhalla IA, Paterson JM, Juurlink DN. Opioid Dose and Drug-Related Mortality in Patients With Nonmalignant Pain. *Arch Intern Med*. 2011;171(7). 10.1001/archinternmed.2011.117 10.1001/archinternmed.2011.117 21482846

[4] Krebs EE, Gravely A, Nugent S (2018). Effect of Opioid vs Nonopioid Medications on Pain-Related Function in Patients With Chronic Back Pain or Hip or Knee Osteoarthritis Pain: The SPACE Randomized Clinical Trial. JAMA..

[5] Abdel Shaheed C, Maher CG, Williams KA, Day R, McLachlan AJ (2016). Efficacy, Tolerability, and Dose-Dependent Effects of Opioid Analgesics for Low Back Pain: A Systematic Review and Meta-analysis. JAMA Intern Med..

[6] Dowell D, Haegerich TM, Chou R (2016). CDC Guideline for Prescribing Opioids for Chronic Pain—United States, 2016. JAMA..

[7] Chou R, Fanciullo GJ, Fine PG (2009). Clinical Guidelines for the Use of Chronic Opioid Therapy in Chronic Noncancer Pain. J Pain..

[8] VA/DoD Clinical Practice Guidelines: Management of Opioid Therapy For Chronic Pain. U.S. Department of Veterans Affairs. Accessed April 14, 2021. http://www.healthquality.va.gov/guidelines/Pain/cot

[9] HHS Guide for Clinicians on the Appropriate Dosage Reduction or Discontinuation of Long-Term Opioid Analgesics. U.S. Department of Health and Human Services. Accessed April 14, 2021. https://www.hhs.gov/opioids/sites/default/files/2019-10/Dosage_Reduction_Discontinuation.pdf

[10] Dowell D, Haegerich T, Chou R (2019). No Shortcuts to Safer Opioid Prescribing. N Engl J Med..

[11] Chua K-P, Kimmel L, Brummett CM (2020). Disappointing Early Results From Opioid Prescribing Limits for Acute Pain. JAMA Surg..

[12] Davis CS, Lieberman AJ, Hernandez-Delgado H, Suba C (2019). Laws limiting the prescribing or dispensing of opioids for acute pain in the United States: A national systematic legal review. Drug Alcohol Depend..

[13] Dave CV, Patorno E, Franklin JM (2019). Impact of State Laws Restricting Opioid Duration on Characteristics of New Opioid Prescriptions. J Gen Intern Med..

[14] Frank JW, Lovejoy TI, Becker WC (2017). Patient Outcomes in Dose Reduction or Discontinuation of Long-Term Opioid Therapy: A Systematic Review. Ann Intern Med..

[15] Frank JW, Levy C, Matlock DD (2016). Patients’ Perspectives on Tapering of Chronic Opioid Therapy: A Qualitative Study. Pain Med..

[16] Perez HR, Buonora M, Cunningham CO, Heo M, Starrels JL (2020). Opioid Taper Is Associated with Subsequent Termination of Care: a Retrospective Cohort Study. J Gen Intern Med..

[17] Matthias MS, Johnson NL, Shields CG (2017). “I’m Not Gonna Pull the Rug out From Under You”: Patient-Provider Communication About Opioid Tapering. J Pain..

[18] Kennedy LC, Binswanger IA, Mueller SR (2018). “Those Conversations in My Experience Don’t Go Well”: A Qualitative Study of Primary Care Provider Experiences Tapering Long-term Opioid Medications. Pain Med..

[19] Posner K, Brown GK, Stanley B, et al. The Columbia–Suicide Severity Rating Scale: Initial Validity and Internal Consistency Findings From Three Multisite Studies With Adolescents and Adults. *Am J Psychiatry*. 2011;168(12):1266-1277. 10.1176/appi.ajp.2011.10111704 10.1176/appi.ajp.2011.10111704 PMC3893686 22193671

[20] DeCuir-Gunby JT, Marshall PL, McCulloch AW (2011). Developing and Using a Codebook for the Analysis of Interview Data: An Example from a Professional Development Research Project. Field Methods..

[21] MacQueen KM, McLellan E, Kay K, Milstein B (1998). Codebook Development for Team-Based Qualitative Analysis. CAM J..

[22] Guest G, Bunce A, Johnson L (2006). How Many Interviews Are Enough?: An Experiment with Data Saturation and Variability. Field Methods..

[23] McNeilage AG, Avery NS, Holliday S, Glare PA, Ashton-James CE. A qualitative trajectory analysis of patients’ experiences tapering opioids for chronic pain. *Pain*. 2021;Publish Ahead of Print. 10.1097/j.pain.0000000000002336 10.1097/j.pain.0000000000002336 33990111

[24] Benintendi A, Kosakowski S, Lagisetty P, Larochelle M, Bohnert ASB, Bazzi AR. “I felt like I had a scarlet letter”: Recurring experiences of structural stigma surrounding opioid tapers among patients with chronic, non-cancer pain. *Drug Alcohol Depend*. Published online March 2021:108664. 10.1016/j.drugalcdep.2021.108664 10.1016/j.drugalcdep.2021.108664 PMC8058315 33757709

[25] Joosten E. Effect of shared decision-making on therapeutic alliance in addiction health care. *Patient Prefer Adherence*. Published online October 2008:277. 10.2147/PPA.S4149 10.2147/ppa.s4149 PMC2770415 19920974

[26] Matthias MS (2020). Opioid Tapering and the Patient-Provider Relationship. J Gen Intern Med..

[27] Hughes HK, Korthuis PT, Saha S (2015). A mixed methods study of patient–provider communication about opioid analgesics. Patient Educ Couns..

[28] Crawley A, Murphy L, Regier L, McKee N (2018). Tapering opioids using motivational interviewing. Can Fam Physician Med Fam Can..

[29] Davis B, Archambault C, Davis K (2019). A patient-centered approach to tapering opioids. J Fam Pract..

[30] Chapter 3—Motivational Interviewing as a Counseling Style. In: *TIP 35: Enhancing Motivation for Change in Substance Use Disorder Treatment*. Substance Abuse and Mental Health Services Administration; 2019. https://store.samhsa.gov/product/TIP-35-Enhancing-Motivation-for-Change-in-Substance-Use-Disorder-Treatment/PEP19-02-01-003. Accessed 18 May 2021

[31] Roper KL, Jones J, Rowland C, Thomas-Eapen N, Cardarelli R (2021). Mixed Methods Study of Patient and Primary Care Provider Perceptions of Chronic Pain Treatment. Patient Educ Couns..

[32] Heiwe S, Lönnquist I, Källmén H (2011). Potential risk factors associated with risk for drop-out and relapse during and following withdrawal of opioid prescription medication. Eur J Pain Lond Engl..

