# Supplementary material for: Patient Perspectives on Improving Patient-Provider Relationships and Provider Communication During Opioid Tapering
Source: J Gen Intern Med. 2022 Jan 6;37(7):1722–8. doi: 10.1007/s11606-021-07210-9 (PMC9130417; doi:10.1007/s11606-021-07210-9)
Supplement: Supplementary file 1 — (DOCX 25.5 kb) [file 11606_2021_7210_MOESM1_ESM.docx]

**Appendix: Changes in Opioid Prescribing Effects (COPE) Qualitative Interview Guide**

**INTRODUCTORY SCRIPT:** Hi, my name is **[Interviewer name]** and I’m an interviewer for this research study.

Thank you for agreeing to be part of this interview. The reason you are here today is because we want to learn about your experiences with opioids. Your input is of great value to us because it may help us improve services for patients undergoing changes in their medications.

We are interested in your honest opinions, whether they are positive or negative. We will not share what you tell us with any of your medical providers or anyone outside of this research study. We will summarize what we learn from you today and make recommendations about how health services can be improved, but we won’t name anyone who made which recommendations or include any information that identifies specific people.

Some of the questions I will ask may deal with sensitive topics and private behaviors and feelings. Please remember that you do not have to answer any questions that make you feel uncomfortable, and you can also stop and leave the interview at any time.

There are no right or wrong answers to any of the questions I will ask today, so please feel free to speak openly and honestly. All ideas are equally valid, and I just want to learn about your experiences and hear your opinions.

As a reminder, we will be recording this interview so I can focus on our discussion without having to take too many notes. No one outside of this study will have access to these recordings and the audio recordings will be deleted from the recorder once uploaded. The recording will be used for the research purposes of this study only. To protect your privacy, please don’t provide any identifying information, such as names. If you do tell us names, we won’t transcribe/include them in transcripts. **Just to confirm, is it OK with you that I record this interview?**

**Do you have any questions before we start?**

**[TURN ON TAPE RECORDER; READ]:** OK, this is [**Interviewer name**] conducting a qualitative interview with **[ID]** in **[site]** on **[date]** at about **[time].**

| **Key terms and concepts** |
| --- |

This study focuses on the experiences of individuals who have undergone an opioid taper. By taper, I mean reducing the dose over time, which may or may not lead to completely stopping taking the medications. Do you have any questions about what that means?

| **SECTION 1: Opioid prescribing history up to taper** |
| --- |

1. To get started, can you tell me about the chronic pain problems you were having that led to being prescribed opioids?

- Specifics of condition (e.g. what, when started)
- Prior medications and self-treatment

1. Tell me about when you were first prescribed opioids.

- What was prescribed, dose

1. How would you describe your initial experience taking opioids for pain?

- Did opioids effectively manage pain
- Comparison to other treatments
- Perceived benefits
- Risks/harms, concerns

1. Tell me about any times that your medical provider changed your opioid medications.

- Reasons for changes, type of changes

1. Tell me about communication with this medical prover. What was decision-making like with this provider?

- How provider communicated/explained changes
- Any treatment monitoring (e.g., urine drug tests, pill counts)
- How monitoring affected relationship/trust
- Ability for patient to raise concerns

1. What was going on in your life when this opioid taper came about? How did the decision to taper your opioids come about?

- Circumstances surrounding taper (including reasons why; e.g., dose no longer effective, “binging” or using faster than prescribed, other/poly-substance use, loss of control, stolen/sold/diverted meds)
- Self-motivated concern or self-awareness of developing SUD

1. How did you initially feel about tapering?

- Fear of inadequately treated pain, withdrawal, ability to control speed of taper
- How concerns were communicated, heard, addressed
- Ability to discuss these concerns with provider
- Self-motivated concern or self-awareness of developing SUD

| **SECTION 2: Taper consequences/benefits** |
| --- |

1. Can you describe what happened when your opioids were tapered? How did you experience chronic pain during the taper?
2. How did you feel during the taper?
   - How was your physical health affected by the taper?
   - How was your mental health affected by the taper?
3. What was your medical care like during and after the taper?

- Pace, frequency of visits
- Communication with provider(s)
- Relationship/trust with provider(s)

1. How did you manage your pain during the/after the taper?

- How provider(s) helped, strategies to manage pain on one’s own
- Knowledge or us of alternative treatments

1. Was there a point during the taper when you felt the worst?
   - How would you describe this pain?
   - What were your feelings and thoughts at this point?
2. Have you had thoughts of harming yourself during or after your taper?
   - Did you ever make a suicide attempt or engage in self-harm?
3. Tell me about any times you considered using non-prescribed substances to deal with your pain on your own.

- What thought processes were like
- Relationship to timing of taper

1. IF YES (Used other substances)
   1. Tell me about your experience using these drugs/medications.
   2. What other substances did you use? (alcohol, marijuana, prescription opioids from other sources, heroin, cocaine, benzodiazepines, gabapentin (Johnnies), other drugs)
   3. Did they help?
   4. How did you begin using these substances? (Influence from friends/family?)
   5. Did you feel that these substances had any potential risks?
   6. Did you share this information or any concerns with your provider?
2. IF NO (Did not use other substances)
   1. Did you ever consider using other substances, or opioids from sources other than your doctor, when your opioid dose was being reduced?
   2. What are the reasons you didn’t use/didn’t consider it?
   3. Did you seek opioid prescribing outside of BMC?
   4. Have you ever considered using non-prescribed or illegal opioids (like heroin)?

| **SECTION 3: Substance use/treatment before, during, and after opioid treatment** |
| --- |

1. Now can you tell me about your use of alcohol or any drugs **before** you began the opioid taper?

- Types and sources of substances used (alcohol, marijuana, prescription opioids from other sources, heroin, cocaine, benzodiazepines, gabapentin (Johnnies), other drugs)

1. What was your alcohol or other drug use like **during** the opioid taper?

- Same as above

1. Tell me about your alcohol or drug use **after** the opioid taper.

- Same as above
- Ways alcohol and other drug use changed over these periods
- Have you ever experienced an overdose?

1. Can you tell me about any experiences you’ve had in mental health treatment?

- [If participant ever received treatment]
  - - Decision to seek treatment (was it offered/recommended by provider or self-initiated)
    - Transition to treatment
    - How helpful treatment is perceived to be
- [If participant never received treatment]
  - - Was it ever offered/recommended
    - Reasons for considering (or not) and not initiating
    - Ongoing substance use

1. Can you tell me about any experiences you’ve had in substance use treatment?

- [If participant ever received treatment]
  - - Decision to seek treatment (was it offered/recommended by provider or self-initiated)
    - Transition to treatment
    - How helpful treatment is perceived to be
- [If participant never received treatment]
  - - Was it ever offered/recommended
    - Reasons for considering (or not) and not initiating
    - Ongoing substance use

| **SECTION 4: Retention and closing** |
| --- |

1. If someone else you know was about to experience an opioid taper, what would you tell them? What advice would you give their medical team?
2. Thank you very much for sharing your experiences with me. Thinking about these experiences, is there anything else you think we should know about opioid tapers?
